# Smoking during pregnancy and gestational diabetes mellitus: a systematic review and meta-analysis

**DOI:** 10.1007/s12020-023-03423-6

**Published:** 2023-06-22

**Authors:** Kleoniki I. Athanasiadou, Stavroula A. Paschou, Evgenia Papakonstantinou, Vasiliki Vasileiou, Fotini Kanouta, Paraskevi Kazakou, Katerina Stefanaki, Georgia N. Kassi, Theodora Psaltopoulou, Dimitrios G. Goulis, Eleni Anastasiou

**Affiliations:** 1 Endocrine Unit and Diabetes Centre, Department of Clinical Therapeutics, Alexandra Hospital, School of Medicine, National and Kapodistrian University of Athens, Athens, Greece; 2 Department of Pediatric Oncology, Ippokratio General Hospital, Thessaloniki, Greece; 3 Department of Endocrinology, Alexandra Hospital, Athens, Greece; 4 Unit of Reproductive Endocrinology, 1st Department of Obstetrics and Gynaecology, Medical School, Aristotle University of Thessaloniki, Thessaloniki, Greece

**Keywords:** gestational diabetes mellitus, GDM, cigarette smoking, tobacco, pregnancy, perinatal

## Abstract

**Purpose:**

To investigate whether maternal cigarette smoking during pregnancy is a risk factor for developing GDM.

**Methods:**

MEDLINE, Scopus, CENTRAL and Google Scholar databases were searched from inception to December 2022 to identify eligible original articles. A systematic review and meta-analysis (weighted data, random-effects model) were performed. The primary outcome was the development of GDM in pregnant women. The results were expressed as odds ratios (OR) with 95% confidence interval (CI) (inverse variance method). Subgroup analysis was planned according to the maternal smoking status and GDM diagnostic criteria. Statistical heterogeneity was checked with the Chi-squared (Chi^2^) test and the I^2^ index was used to quantify it. The studies were evaluated for publication bias.

**Results:**

Thirty-five studies, including 23,849,696 pregnant women, met the inclusion criteria. The pooled OR of smoking during pregnancy compared with non-smoking (never smokers and former smokers) was 1.06 (95% CI 0.95–1.19), p = 0.30; I^2^ = 90%; Chi^2^ = 344; df=34; p < 0.001. Subgroup analysis was performed according to the two-step Carpenter-Coustan diagnostic criteria, due to the high heterogeneity among the other applied methods. The pooled OR for the Carpenter-Coustan subgroup was 1.19 (95% CI 0.95–1.49), p = 0.12; I^2^ = 63%; Chi^2^ = 27; df=10; p < 0.002. Further subgroup analysis according to maternal smoking status was not performed due to missing data.

**Conclusion:**

There is no evidence to support an association between maternal cigarette smoking during pregnancy and the risk for GDM. Universally accepted diagnostic criteria for GDM must be adopted to reduce heterogeneity and clarify the association between smoking and GDM.

## Introduction

Gestational diabetes mellitus (GDM) is a hyperglycemic disorder first recognised during the second or third trimester of pregnancy, with major perinatal complications and long-term metabolic risks for the mother and the offspring [1]. During the last decades, women have tended to give birth in more advanced age and be more overweight, and as a result GDM prevalence is on the rise [2]. There are several factors predisposing to GDM, with advanced maternal age ( ≥ 35 years), maternal obesity (body mass index ≥ 30 kg/m^2^) and history of GDM in previous pregnancy being the most prominent [3]. Moreover, weight gain during pregnancy, family history of diabetes, polycystic ovary syndrome (PCOS), ethnicity (Asian, Asian American, African American, Native American, Pacific Islander, Latin), and achievement of pregnancy with Assisted Reproductive Technologies (ART) have been associated with GDM development [4, 5]. Cigarette smoking is a considerable threat to public health in both developed and developing countries, associated with all the leading causes of death [6]. Smoking is a modifiable risk factor for major cardiovascular events (MACE), cancer, type 2 diabetes mellitus (T2DM), and chronic obstructive pulmonary disease (COPD) [7, 8]. According to a meta-analysis, the global prevalence of smoking during pregnancy is 1.7%, with the highest being in the European region (8.1%) [9]. Smoking during pregnancy is related to placenta abruption, placenta previa, stillbirth, preterm premature rupture of membranes (PPROM) preterm delivery, pre-eclampsia, intrauterine growth restriction (IUGR), and congenital anomalies [10–12]. Tobacco smoking alters placental immunoregulation and function, and damage the feto-maternal interface at a cellular level [13]. Although cigarette smoking is a devasting and potentially destructive habit for the growing fetus, it is unclear whether it predisposes the mother to GDM development [14]. Several studies indicate passive or “secondhand” smoking as a risk factor for GDM development [15–17]. Two systematic reviews and meta-analyses concluded that there is no association between maternal smoking during pregnancy and GDM [18, 19]. As many relevant original studies have been published since 2017, the topic is worth a meta-analysis update.

## Methods

### Search strategy

The present study was conducted according to the recommendations of PRISMA guidelines (Preferred Reporting Items for Systematic Review and Meta-Analyses) [20]. The study’s protocol was enrolled in PROSPERO (registration number: CRD42022378857). A literature search in MEDLINE (PubMed), Scopus (Elsevier), CENTRAL (Cochrane Central Register of Controlled Trials), and Google Scholar databases was performed independently by two researchers (KIA and EP) from inception to December 28th, 2022.

### Eligibility criteria

Eligible for inclusion were considered all the original studies [observational (cohort, cross-sectional, case-control) or interventional (randomized controlled trials)] written in the English language assessing the association of maternal cigarette smoking during pregnancy with the risk of GDM development. Studies involving pregnant women with pre-existing type 1 or T2DM, non-pregnant populations, pediatric, adolescent, or male populations, as well letters to the editor, editorials, case reports and case series were excluded.

### Outcomes

The primary outcome of interest was the development of GDM in pregnant women smoking during pregnancy. The first arm included the active smokers and the second the non-smokers (never-smokers and former smokers). Former smokers ceased smoking at the beginning of gestation.

### Study selection

The study selection was performed by two researchers (KIA and EP) independently; disagreements were resolved with the contribution of a third researcher (SAP), leading to consensus. The study selection was guided by the eligibility criteria and outcome of interest. The identified studies were imported to a systematic review software platform (COVIDENCE). Their title and abstract were screened for relevance, and followingly the full texts of selected studies were screened further for the final selection. Finally, the references of selected articles were also screened for the detection of more eligible studies.

### Data extraction

The data extraction was performed in a pre-designed Microsoft Excel^®^ sheet by two researchers (KIA and EP) that collected data independently on the name of the first author, the paper’s title, the year of the paper’s publication, the number of participants, the study’s type, the journal of publication, the country of origin, the study’s duration, the primary outcome, the study’s conclusion, the number of women in each subgroup (never-smokers, former and current smokers), the number of GDM cases in each subgroup, the diagnostic method of GDM, the confounders adjusted in each study, and the study’s conclusion. Any disagreements were resolved by a third researcher (SAP).

### Statistical analysis

Statistical analysis was performed using Review Manager (RevMan) software (version 5.4.1 for Mac OS), as recommended by the Cochrane Collaboration. Dichotomous data were analyzed using an odds ratio (OR) and a 95% confidence interval (CI), according to the Inverse Variance method. The level of significance was set at 5%. The data were combined using the random-effects model because of the high heterogeneity among the included studies. The presence of heterogeneity was checked with the Chi-squared (Chi^2^) test (p-value < 0.1) and was quantified with the I^2^ Higgins test (0–40% non-significant/low heterogeneity, 40–50% moderate heterogeneity, ≥50% significant/high heterogeneity) [21]. A sensitivity analysis was performed to explain the high heterogeneity among studies. By removing one study at a time and recalculating the OR, the effect of each study on the summary estimate was observed. In the primary analysis, the effect of smoking versus non-smoking (never-smokers and former smokers) during pregnancy was investigated. In the subgroup analysis, the effect of GDM diagnostic method on the main outcome was analyzed.

### Risk of bias

The risk of bias was assessed by two independent researchers (KIA and EP) with the 9-star Newcastle-Ottawa Scale (NOS) for cohort and case control studies (1–5 low quality, 6–9 high quality), and with the revised Joanna Briggs Institute (JBI) scale for cross-sectional studies (1–5 low quality, 6–8 high quality) [22, 23]. Publication bias was assessed with the construction of a funnel plot [24].

## Results

### Study selection

**Fig. 1:**
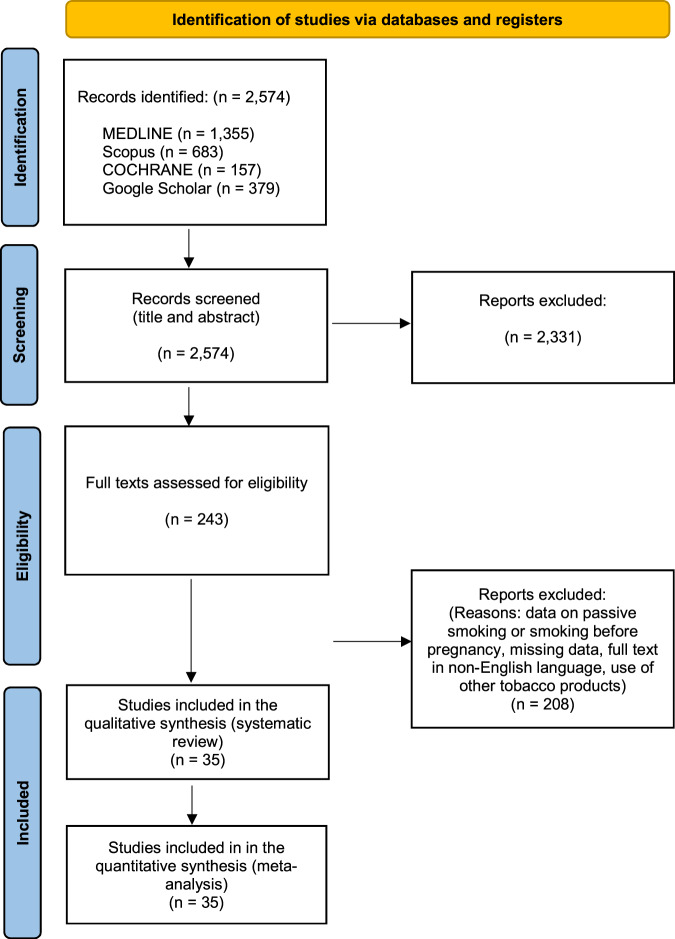
PRISMA flowchart of studies for inclusion in the systematic review and the meta-analysis

With the initial search 2,574 articles were retrieved. After title and abstract screening, 243 studies remained for full text assessment. Finally, 35 articles were included in the present systematic review and meta-analysis. The PRISMA (Preferred Reporting Items for Systematic Reviews and Meta-analyses) flowchart of selected studies is presented in Fig. 1.

### Study characteristics

The characteristics of the included studies, published between 1992 and 2022, are summarized in Table 1.

**Table 1:** Characteristics of the studies included in the systematic review.

Study ID	Study type	Study population	Study duration	Country	Effect estimate	Increased GDM risk	Potential confounders	GDM diagnosis	Quality score	Conclusions
Varela et al. 2022 [16]	Case-control	1,262	3^2/12^ y	Spain	OR 1.22, 95% CI 0.71, 2.10	**(** + **)**	maternal age, BMI	50-g OGTT	5	Slightly increased risk of GDM in women who smoked during pregnancy.
Feferkorn et al., 2021 [31]	Case-control	14,882	11 y	USA	aOR 1.50, 95%CI 1.01, 2.23	**(** + **)**	maternal obesity, chronic hypertension	two-step Carpenter-Coustan	8	Significant association between smoking and GDM in women with PCOS.
Kim et al., 2021 [32]	Cohort	417,139	5 y	Korea	aOR 1.07, 95%CI 1.02, 1.12	**(** + **)**	maternal age, BMI, waist circumference, FPG, location, income, smoking, drinking, exercise	two-step Carpenter-Coustan	6	Current smokers at greater risk for GDM than non-smokers.
Chanda et al., 2020 [33]	Cross-sectional	1,212	2 m	India	aOR 1.61, 95%CI 1.10, 2.36	**(** + **)**	n/a	75-g OGTT (Indian variation)	4	Smoking is a significant and independent predictor of GDM.
Masalin et al., 2020 [34]	Cohort	4,111	7 y	Finland	aOR 1.65, 95%CI 1.09, 2.50	**(** + **)**	maternal weight gain after smoking cessation in early pregnancy, excessive weight gain, socioeconomic deprivation, thinner and shorter mothers	75-g OGTT (Finnish variation)	6	Smoking during pregnancy associated with an increased risk for GDM.
Bar-Zeev et al., 2019 [35]	Cohort	222,408	7 y	USA	aOR 1.46, 95%CI 1.25, 1.71	**(** + **)**	maternal age, race/ethnicity, pre-pregnancy BMI, gestational weight gain	self-reported	7	Prenatal smoking associated with higher odds of GDM.
Konstantakou et al., 2019 [36]	Cohort	7,437	6 y	Greece	OR 1.02, 95%CI 0.87, 1.19	**(-)**	maternal BMI, gestational weight gain	100-g OGTT	8	No correlation between smoking and GDM risk.
Garmendia et al., 2019 [37]	Cohort	86,362	14 y	Chile	aOR 0.83, 95%CI 0.76, 0.91	**(-)**	n/a	75-g OGTT (Chilean variation)	6	Smoking negatively associated with GDM.
Sirico et al., 2019 [38]	Cohort	603	3 y	Italy	aOR 2.34, 95%CI 1.03, 5.33	**(** + **)**	maternal age, BMI, fetal heart rate	75-g OGTT (0’, 60’, 120’)	5	Maternal cigarette smoking independently associated with GDM.
Badon et al., 2017 [39]	Cohort	3,005	13 y	USA	aOR 0.58, 95%CI 0.33, 1.02	**(** + **)**	maternal age, race, nulliparity, pre-pregnancy BMI, physical activity, smoking	two-step Carpenter-Coustan	7	Non-smoking significantly associated with reduced risk of GDM.
Collier et al., 2017 [40]	Cohort	47,290	32 y	Scotland	aOR 0.70, 95%CI 0.60, 0.82	**(-)**	maternal age, BMI, parity, smoking, Scottish index of multiple deprivation (SIMD)	retrieved from registry record	7	GDM negatively associated with smoking.
Xu et al., 2017 [41]	Cross-sectional	2,345	single point in time	China	OR 0.89, 95%CI 0.28, 2.83	**(-)**	maternal age, BMI, income, trimester of pregnancy, hypertension	self-reported	5	Maternal smoking and second-hand smoke exposure was not associated with an increased risk of GDM in this study.
Erem et al., 2015 [42]	Cross-sectional	815	single point in time	Turkey	OR 0.37, 95%CI 0.02, 6.24	**(-)**	maternal age, BMI, smoking, weight gain, GDM history, family history of diabetes	two-step Carpenter-Coustan	7	GDM was less frequent in current smokers than in former smokers and non-smokers.
Moore Simas et al., 2014 [43]	Cohort	3,029	6 y	USA	aOR 0.47, 95% CI 0.23, 0.96	**(-)**	maternal age, parity, study site	100-g OGTT	6	Smoking during pregnancy associated with a reduction in the odds of GDM.
Huy et al., 2012 [44]	Cohort	650,232	10 y	Germany	aOR 1.11, 95%CI 1.02, 1.21	**(-)**	maternal age, BMI, parity, multiple pregnancy, age at birth of child, weight increase during pregnancy, mother’s nationality, profession	retrieved from maternity record	6	The risk for GDM associated with age, BMI, and social class of pregnant women as well as with multiple pregnancies.
Lagerros et al., 2012 [45]	Cohort	323,083	31 y	Sweden	OR 1.10, 95%CI 0.90, 1.34	**(-)**	maternal age, birth weight, BMI, height, parity, education, smoking	75-g OGTT (0’, 120’)	7	Smoking did not affect the risk for GDM.
Hosler et al., 2011 [46]	Cross-sectional	2,690	2 y	USA	aOR 0.89, 95%CI 0.53, 1.49	**(-)**	maternal age, race/ethnicity, pre-pregnancy BMI, hypertension, smoking exposure, education, parity, gestation at first visit for prenatal care	self-reported or GDM indicated on birth certificate	7	Smoking exposure had no association with GDM and did not show a dose-response pattern.
Haskins et al., 2010 [47]	Cohort	1,006	3^3/12^ y	USA	aOR 0.38, 95%CI 0.13, 1.11	**(-)**	maternal age, BMI, gestational weight gain, parity, education	50-g OGTT	7	No increased risk of abnormal glucose tolerance associated with smoking during pregnancy.
Roelands et al., 2009 [48]	Cohort	21,207,981	5 y	USA	OR 0.90, 95%CI 0.81, 1.00	**(-)**	n/a	ICD-9 codes from discharge records	6	Smokers were less likely to experience GDM.
Rauh-Hain et al., 2008 [49]	Cohort	23,056	8^4/12^y	USA	aOR 1.05, 95%CI 0.70, 1.58	**(-)**	maternal age, race/ethnicity, BMI, maximal systolic and diastolic blood pressure, smoking, parity	two-step Carpenter-Coustan	8	Smoking during pregnancy not associated with elevated risk for GDM.
Radesky et al., 2008 [50]	Cohort	1,733	n/a	USA	OR 1.17, 95%CI 0.27, 5.07	**(** + **)**	maternal age, pre-pregnancy BMI, race/ethnicity, history of GDM, family history of diabetes, smoking	two-step Carpenter-Coustan	7	Smoking during pregnancy identified as a risk factor for GDM.
Wendland et al., 2008 [51]	Cohort	4,766	4 y	Brazil	aOR 0.69, 95%CI 0.50, 0.95	**(-)**	maternal age, parity, pre-eclampsia, smoking, skin colour, study centre, waist circumference	75-g OGTT (0’, 120’)	8	GDM prevalence inversely associated with smoking during pregnancy in nulliparous women, independent of adiposity.
Cosson et al., 2006 [52]	Cohort	1,679	1 y	France	OR 0.65, 95% CI 0.43, 0.98	**(-)**	maternal age, pregravid BMI, parity, ethnicity	75-g OGTT (0’, 120’)	8	Smoking during pregnancy not identified as a risk factor for GDM.
Östlund et al., 2004 [53]	Cohort	430,852	5 y	Sweden	aOR 0.63, 95%CI 0.59, 0.67	**(-)**	maternal age, parity, smoking habits, chronic hypertension, and pre-existing kidney disease	75-g OGTT (0’, 120’)	7	Smoking during pregnancy associated with decreased risk for GDM.
England et al., 2004 [54]	Cohort	3,774	3 y	USA	aOR 1.90, 95%CI 1.00, 3.61	**(** + **)**	maternal age, race, ethnicity, BMI, education, previous pregnancy loss at ≤20 weeks, private health insurance, study centre, gestational age	two-step Carpenter-Coustan	7	Women who smoked at increased risk of GDM.
Terry et al., 2003 [55]	Cohort	212,190	9 y	Sweden	OR 1.10, 95% CI 0.81, 1.49	**(-)**	maternal age, cohabitation with the child’s father, maternal height, BMI in the second pregnancy, interpregnancy interval, smoking and GDM in the first pregnancy	75-g OGTT (0’, 120’)	7	Cigarette smoking not associated with an increased risk for GDM.
Wolf et al., 2003 [56]	Case-control	137	3 y	USA	OR 0.85, 95% CI 0.39, 1.86	**(-)**	maternal age, race, ethnicity, smoking, parity, blood pressure, gestational age	two-step Carpenter-Coustan	6	No difference in age, smoking, parity, and blood pressure among the case and control subjects.
Yang et al., 2002 [57]	Cohort	9,471	1 y	China	aOR 7.82, 95% CI 1.73, 35.35	**(** + **)**	maternal age, BMI, weight gain, education, average household income, pregnancy-induced hypertension, gestational weeks at GCT, diabetes in first-degree relatives and in other relatives, maternal GDM, parity, abortion, history of serious pregnancy outcomes, smoking, alcohol drinking	ingestion of 200 ml of 25% glucose solution; if Glu >140 mg/dl, then 75g-OGTT	7	Women who smoked or had a short stature more likely to develop GDM than their counterparts.
Innes et al., 2002 [58]	Case-control	23,395	5 y	USA	aOR 1.15, 95%CI 0.88, 1.50	**(-)**	maternal age, BMI, race, education, height, weight gain, birth weight, gestational age, employment status, maternal diabetes	ICD-9 codes from discharge records	7	Maternal smoking during pregnancy not associated with increased risk for GDM.
Bo et al., 2001 [59]	Cohort	504	1^8/12^y	Italy	OR 1.58, 95% CI 1.02, 2.45	**(-)**	maternal age, BMI, familial diabetes, height, percentage of saturated fat, gestational age	two-step Carpenter-Coustan	7	No association between smoking habits and glucose abnormalities in pregnancy.
Xiong et al., 2001 [60]	Cohort	111,419	7 y	Canada	aOR 0.96, 95%CI 0.87, 1.06	**(-)**	maternal age, parity, maternal weight, smoking, alcohol use, history of neonatal death, prematurity, major fetal anomaly, caesarean section	100-g OGTT	6	No association of risk factors, including multiparity, maternal smoking, leaner stature, history of premature delivery and major fetal anomaly, with GDM.
Zarén et al., 2000 [61]	Cohort	1,339	2^3/12^y	Sweden	OR 1.97, 95% CI 0.93, 4.18	**(** + **)**	maternal age, pre-pregnancy body weight, pregnancy weight gain, late gestational serum ferritin or hemoglobin levels, maternal body habitus	75-g OGTT (0’, 120’)	7	Smoking in pregnancy affects parameters of glucose homeostasis in the direction of GDM.
Joffe et al., 1998 [62]	Cohort	3,689	n/a	USA	OR 2.70, 95% CI 1.58, 4.61	**(** + **)**	maternal BMI, race, clinical centre	two-step Carpenter-Coustan	5	Compared with women with normal glucose tolerance, women with abnormal glucose tolerance were likelier to smoke.
Solomon et al., 1997 [63]	Cohort	14,613	5 y	USA	aOR 1.56, 95%CI 1.25, 1.93	**(** + **)**	maternal age, BMI, family history of diabetes, ethnicity, parity, pre-gestational physical activity	self-reported	6	Cigarette smoking predicted increased GDM risk.
Berkowitz et al., 1992 [64]	Cohort	10,187	3 y	USA	OR 0.82, 95% CI 0.54, 1.25	**(-)**	maternal age, pre-pregnancy weight, family history of diabetes	two-step Carpenter-Coustan	7	No association of GDM and cigarette smoking.

*BMI* Body Mass Index, *CI* confidence interval, *FPG* Fasting Plasma Glucose, *GDM* gestational diabetes mellitus, *n/a* non-applicable, *OGTT* Oral Glucose Tolerance Test, *(a)OR* (adjusted) odds ratio

### Data synthesis (meta-analysis)

**Fig. 2:**
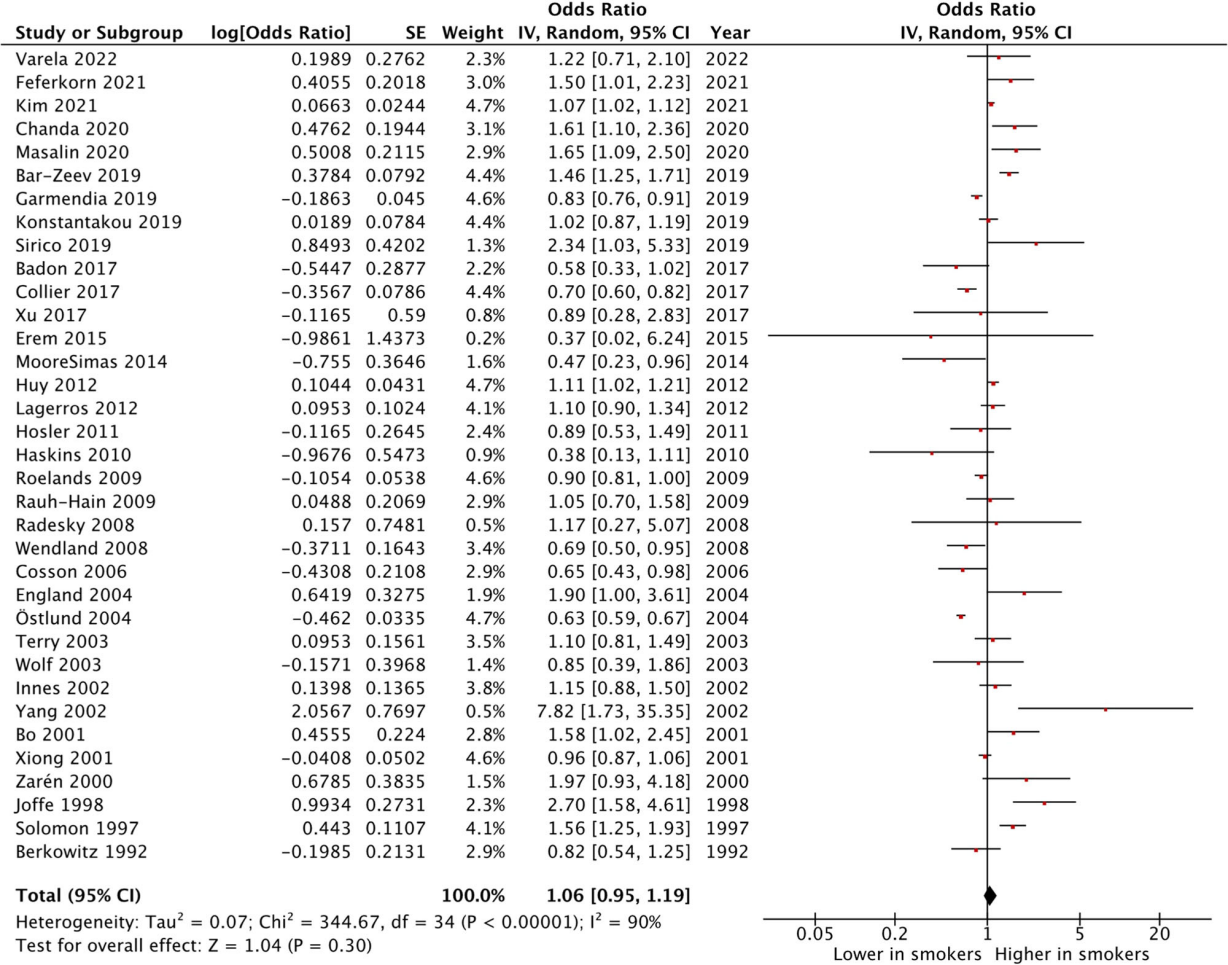
Meta-analysis of primary outcome

The results of the meta-analysis performed are summarized in Fig. 2.

#### GDM in smokers vs. non-smokers

OR 1.06 (95% CI 0.95–1.19, p = 0.30), indicating no significant association between cigarette smoking during pregnancy and GDM development. There was high heterogeneity (I^2^ = 90%; Chi^2^ = 344; df = 34; p < 0.001).

### Subgroup analysis

**Fig. 3:**
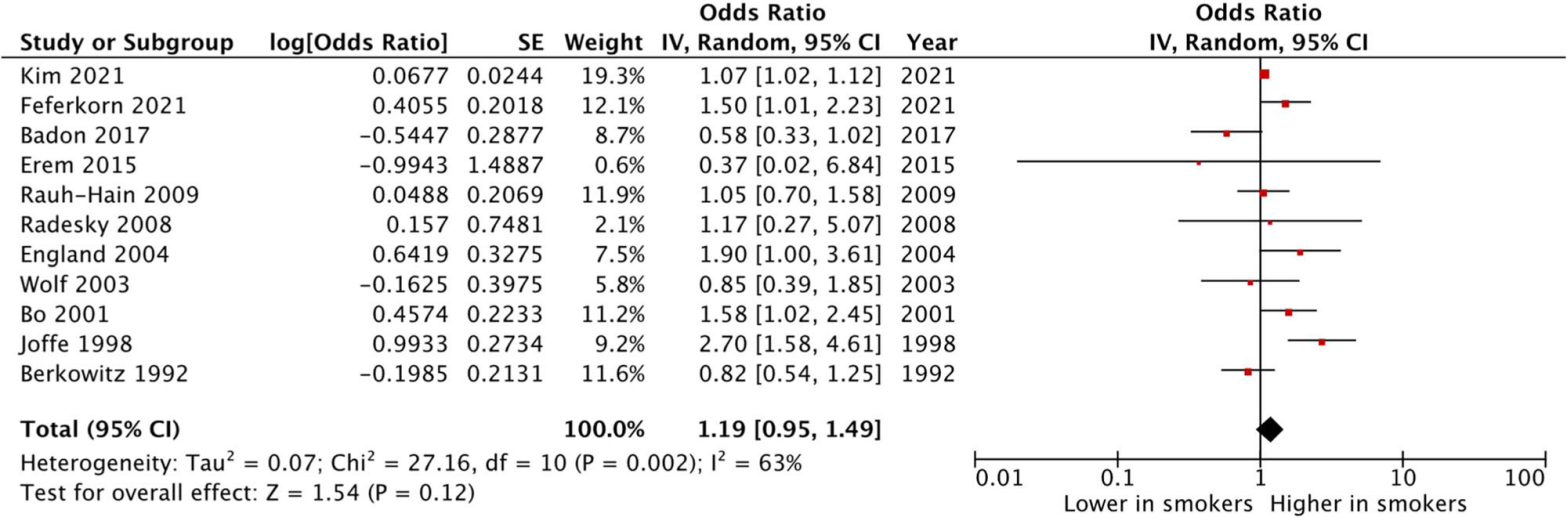
Subgroup analysis (two-step Carpenter-Coustan diagnostic criteria)

Due to the high heterogeneity in the definition of GDM diagnostic methods, we proceeded to subgroup analysis of the studies using the two-step Carpenter-Coustan criteria. The results of subgroup analysis (Fig. 3) did not show an increase in the odds of GDM development in women smoking during pregnancy (OR 1.19, 95% CI 0.95–1.49, p = 0.12; I^2^ = 63%; Chi^2^ = 27; df = 10; p < 0.002) [25]. The heterogeneity was reduced in this subgroup analysis.

Additionally, we performed further subgroup analysis between the studies that provided: adjusted OR (Fig. 5), unadjusted OR (Fig. 6). Moreover, we performed subgroup analysis between the studies that provided adjusted OR and had considered similar confounders, such as: maternal age (Fig. 7), and BMI (Fig. 8). The results were similar with our initial analysis (OR ranging between 0.96–1.12), indicating that the unadjusted results and the various confounders did not distort our final conclusion.

### Sensitivity analysis

High heterogeneity was observed. Removing each study, no significant changes in the effect estimate and I^2^ index were observed.

### Risk of bias

**Fig. 4:**
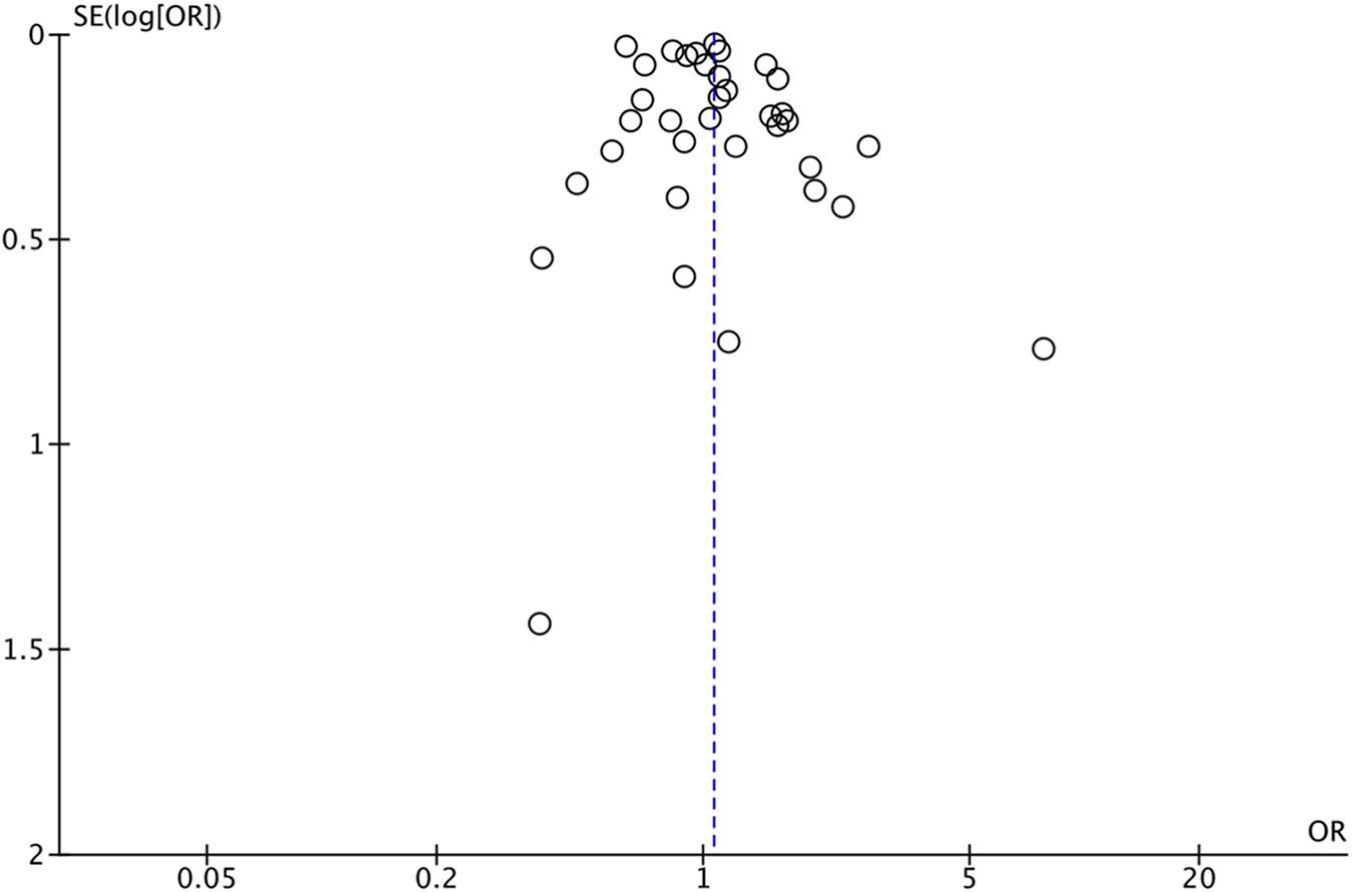
Funnel plot

The quality scores using NOS and the revised JBI scales are summarized in Table 1. Most of the studies were of high quality, and the quality scores ranged from 4 to 8 stars (5–8 for cohort and case-control studies, and 4–7 for cross-sectional). The main reason for downgrading the quality score was missing information on maternal smoking exposure. Publication bias was assessed with the funnel plot presented in Fig. 4. The funnel plot was symmetrical indicating low publication bias.

## Discussion

The present systematic review and meta-analysis aimed to investigate the association between maternal cigarette smoking during pregnancy and the risk for GDM development. There is limited epidemiologic evidence supporting cigarette smoking as a risk factor for GDM. The present data synthesis of 35 studies concluded that maternal smoking during pregnancy is not associated with an increased risk for GDM (OR 1.06, 95% CI 0.95–1.19), a result consistent with the findings of the previous meta-analyses. To our knowledge, this is the third systematic review and meta-analysis (SRMA) on the topic, as there are two SRMAs published in 2008 and 2018 [18, 19]. Wendland et al. published the first relevant systematic review, including 12 studies, and concluded that there was no association between smoking during pregnancy and GDM (OR 1.03, 99% CI 0.85–1.25) [18]. Wang et al. meta-analysis, including 12 studies, found that there was no significant association between cigarette smoking during pregnancy and GDM development (OR 0.98, 95% CI 0.88–1.10) [19].

The present study is a meta-analysis of observational studies, and the potential confounders were acknowledged in Table 1. Results were primarily adjusted for the following confounders: maternal age, BMI, gestational weight gain, parity, race/ethnicity, GDM in a previous pregnancy, hypertension, education, and smoking status. While there was high heterogeneity between studies, sensitivity analysis did not show any influence of a single study on the overall result.

Among the including studies, there were controversial results about the smoking effect on GDM development. Fourteen studies showed an association between smoking during pregnancy and GDM, while 21 studies did not reveal any association (Table 1). However, only 17 studies had significant results.

In the subgroup analysis, an important reduction of heterogeneity was observed when two-step Carpenter-Coustan criteria were used for GDM diagnosis. The wide variations in GDM diagnostic methods and the need for universal GDM screening of pregnant women according to official guidelines are issues that should be urgently resolved.

The HAPO study (Hyperglycemia and Adverse Pregnancy Outcomes) altered the diagnostic approach of hyperglycemia in pregnancy [26]. Followingly, the International Association of Diabetes and Pregnancy Subgroup (IADPSG) established the 2-h 75-g OGTT, a detection strategy that increased the diagnostic prevalence of GDM, to protect mothers and fetuses from the devastating implications of overt hyperglycemia [12, 27].

As tobacco smoking during pregnancy may have detrimental implications for mother and fetus, women are encouraged to avoid it. Nevertheless, the actual mechanisms involved in the association of smoking and GDM manifestation need clarification. Nicotine increases cortisol, catecholamines and growth hormone secretion, hormones that counteract the actions of insulin, contributing to insulin resistance [28]. Pregnancy is a state of increased insulin resistance, owing to placental hormones like the human placental lactogen (hPL) and placental growth hormone, that support the growing fetus [29].

Although women are strongly encouraged to cease smoking during gestation, it was unexpected that some studies showed an association between smoking cessation and increased risk for GDM. A possible cause is that smoking cessation leads to increased caloric consumption and weight gain, a risk factor for GDM. Moreover, changes in adipocytes metabolism and cumulative exposure to smoking before cessation are associated with a greater risk for GDM in women who cease smoking upon gestation [30]. In the present study, we did not proceed to subgroup analysis between smokers and former smokers due to a lack of data.

Smoking results in a toxic environment of inflammatory and oxidative stress, undermining the health of mothers and fetuses. Although policies have been adopted for the prevention of active maternal smoking during pregnancy, exposure to tobacco through passive smoking from partners or/and family remains a threat to pregnant women [15]. The exposure of pregnant women to tobacco might be underestimated, as passive smoking is underreported (high recall and reporting bias); only a limited number of studies consider its impact on maternal outcomes. Passive smoking is an independent risk factor for GDM development in nulliparous women highlighting the need for prompt and intensive health education of pregnant women and their partners/families [15–17]. Preventive strategies should be implemented. In the present study, passive smoking was not independently evaluated, as none of the included studies [except for three (Hosler 2011, Xu 2017, Varela 2022)] provided relevant data.

The current study presents several strengths and certain limitations. Regarding strengths, the meta-analysis included 23,849,696 subjects and 35 studies published between 1992–2022 and conducted in 16 countries from almost every continent (e.g., USA, Canada, Brazil, Sweden, France, Italy, India, Korea, China), covering a representative population. Moreover, it is the third systematic review and meta-analysis evaluating the association between maternal cigarette smoking and the risk of GDM. Nine original studies were published since 2018 and incorporated in the present meta-analysis, along with several older studies.

In terms of limitations, the present systematic review and meta-analysis consists of 35 observational studies, as they were only available in the literature. The only study type that can prove a causation between a risk factor and a certain condition is the randomized controlled trial; nevertheless, it is unethical to conduct a trial with smoking intervention in pregnant women, as it would pose mothers and fetuses at a definitive risk. In all meta-analyses of observational studies, the confounding factors have to be identified and considered for a safe conclusion to be reached. Unfortunately, many of the included studies did not assess the potential confounders and presented unadjusted data, while the corresponding authors did not respond to our request for providing further data. However, subgroup analyses to evaluate the effect of unadjusted results on the final conclusion were conducted; the pooled outcome remained unchanged. Another limitation is that subgroup analysis according to tobacco exposure levels and smoking status was not performed, due to a lack of relevant data. Finally, there was high heterogeneity among the studies, which could be attributed to major differences in participants’ demographic characteristics, smoking assessment, and GDM diagnostic methods.

In conclusion, there is no evidence to suggest an association between maternal cigarette smoking during pregnancy and the development of GDM. However, further studies evaluating the impact of passive smoking and other potential confounding factors on GDM development should be performed. It is imperative, though, that the exposure of pregnant women to cigarette smoke should be limited even from the preconception period through investment in awareness policies, as it creates a toxic environment for the fetomaternal unit. GDM diagnostic criteria deserve a global consensus to establish a common ground on early identification and treatment.

### Supplementary Information

Supplementary material
