# Supplementary material for: Smoking during pregnancy and gestational diabetes mellitus: a systematic review and meta-analysis
Source: Endocrine. 2023 Jun 22;82(2):250–62. doi: 10.1007/s12020-023-03423-6 (PMC10543648; doi:10.1007/s12020-023-03423-6)
Supplement: Supplementary file 1 — Supplementary material [file 12020_2023_3423_MOESM1_ESM.docx]

# Supplementary material


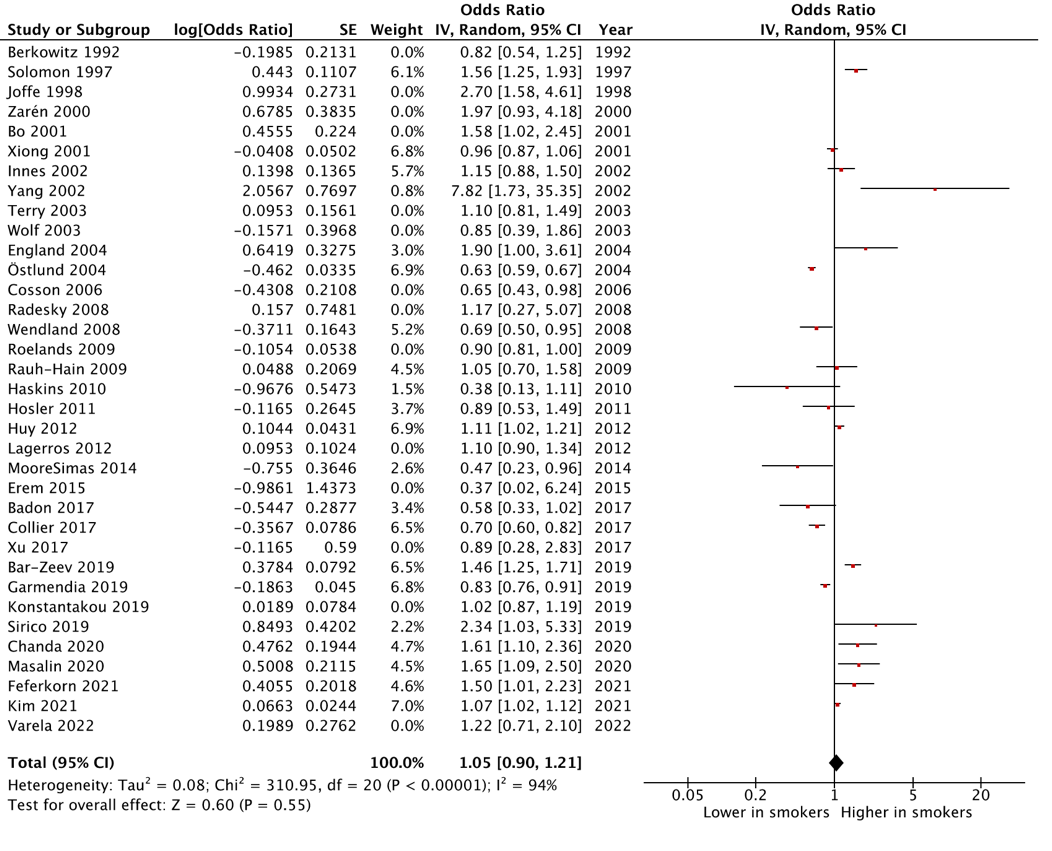


**Fig.5** Subgroup analysis (studies providing adjusted OR)

*
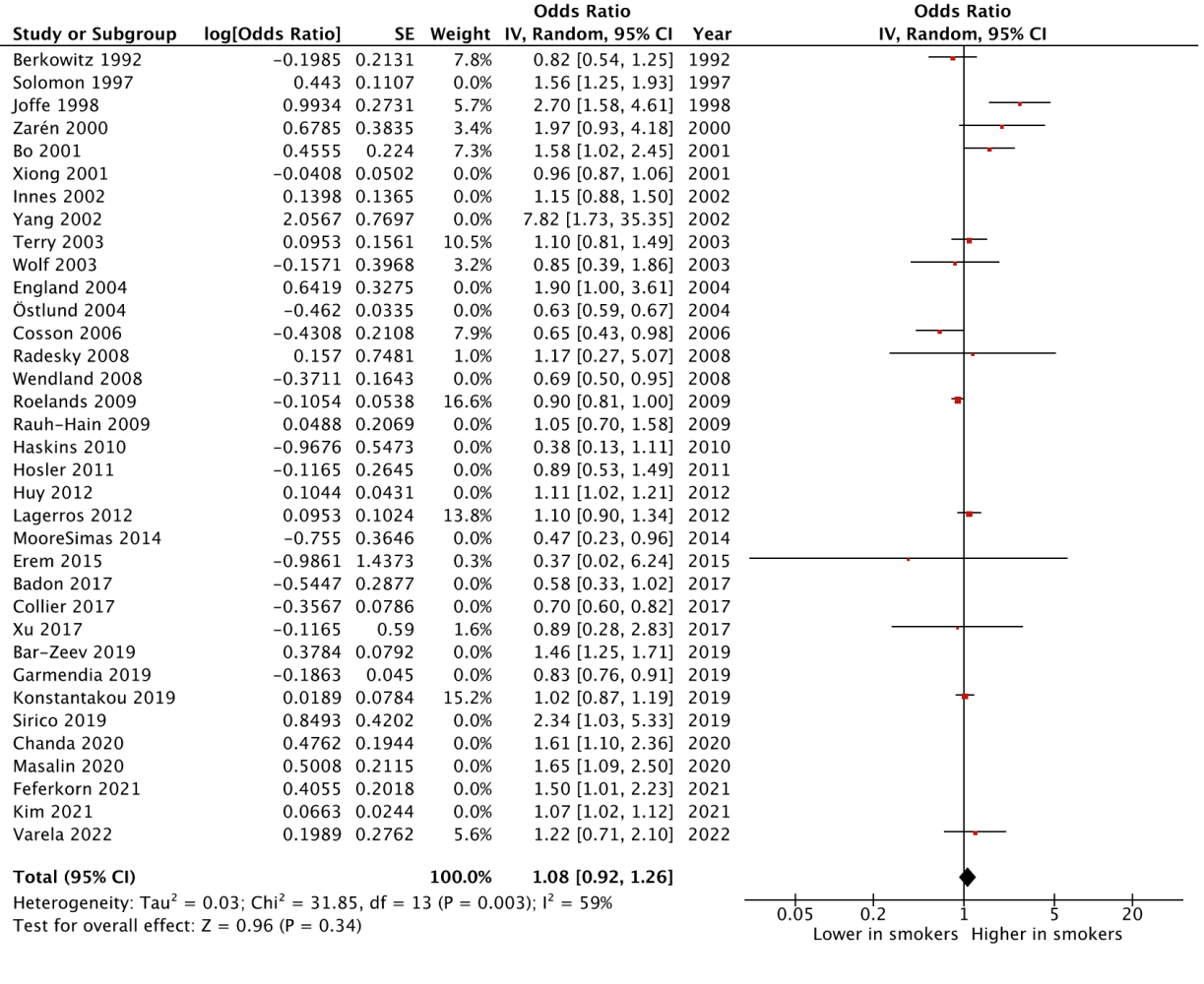
*

**Fig.6** Subgroup analysis (studies providing unadjusted OR)

**
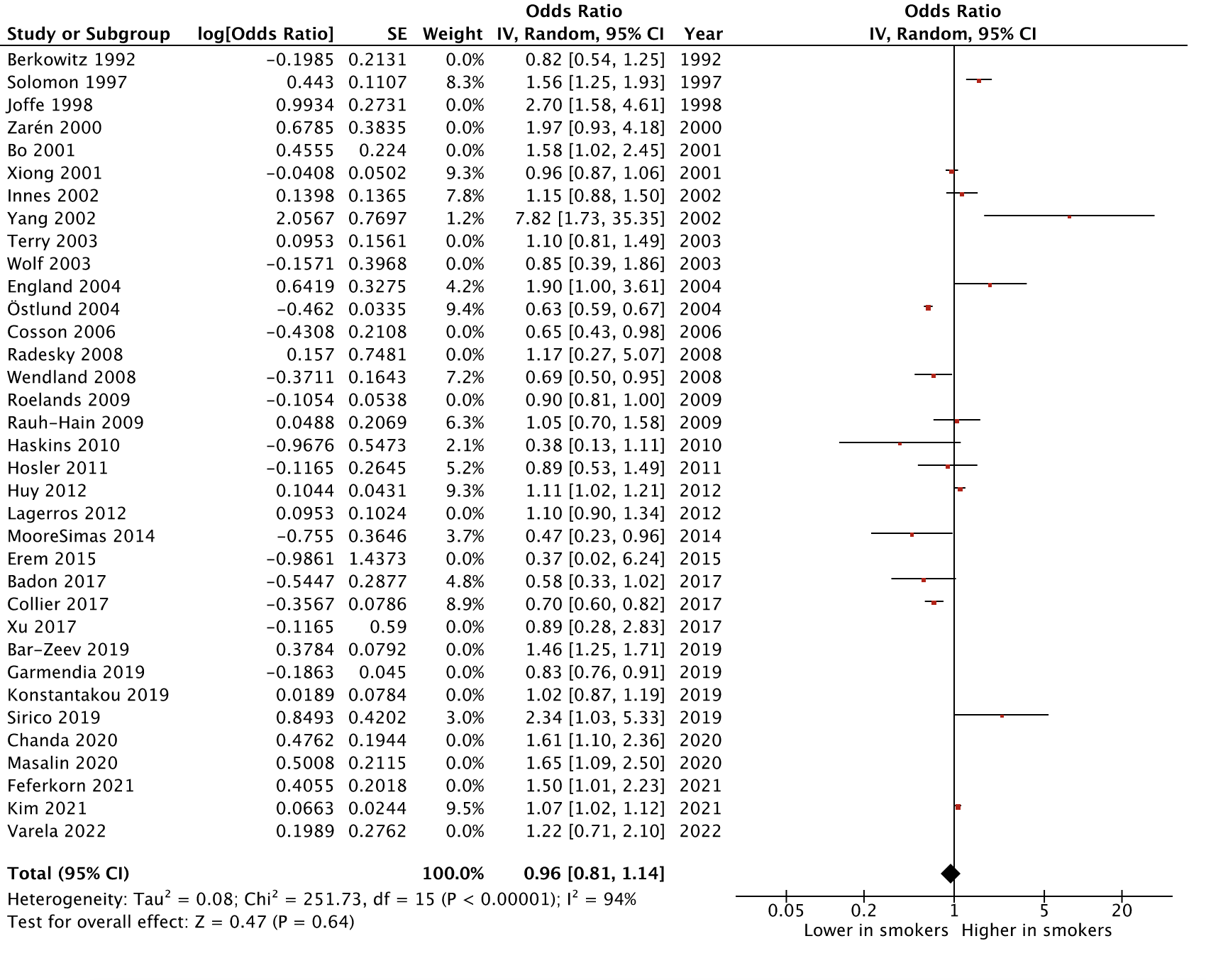
**

**Fig.7** Subgroup analysis (studies providing adjusted OR considering the confounder maternal age)

**
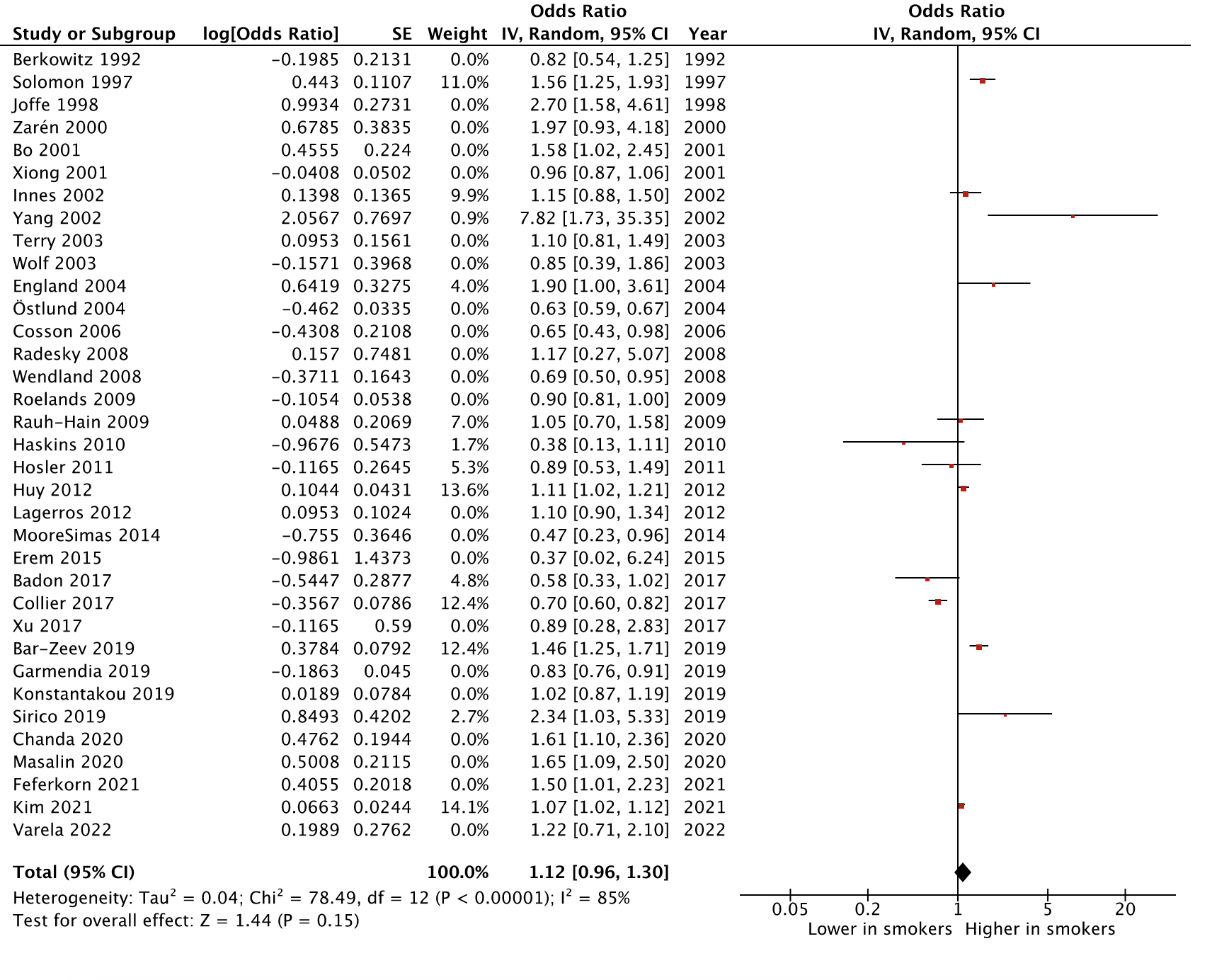
**

**Fig.8** Subgroup analysis (studies providing adjusted OR considering the confounder maternal BMI)
